# Violence against doctors in Nepal: a growing crisis demanding urgent actions

**DOI:** 10.1016/j.lansea.2024.100407

**Published:** 2024-04-10

**Authors:** Sangam Shah, Kiran Paudel, Ashutosh Kashyap

**Affiliations:** a Tribhuvan University, Institute of Medicine, Maharajgunj, 44600, Nepal; b Department of Allied Health Sciences, University of Connecticut, Storrs, Connecticut, USA

Violence against doctors has escalated in Nepal recently, leading to nation-wide protests and strikes from doctors.^1^ Within a month of the agreements with the government, we saw yet another incident of violence at a tertiary care center in the heart of the capital.^2^ This trend is worrying as it jeopardises the well-being of doctors and threatens the integrity of Nepal's healthcare system, which is already in shambles, with a record number of doctors leaving every year amidst an already awful doctor to patient ratio of 1:150,000 in rural Nepal.^3^

With violence ranging from verbal abuse to physical assaults,^4^ patients and their relatives have left no opportunity to thrash doctors and vandalise hospitals in recent times in Nepal. These assaults have not only occurred in heat of the moment situations, but have also taken place as planned crimes, months following patient's discharge or referral. Similar assaults have also led to cold-blooded murders of doctors at workplace in India.^5^ According to a study by Bhusal and colleagues,^6^ about 45.5% of the doctors had experienced some sort of workplace violence in the past. Public have become desensitised to such incidents and there is a perception that doctors deserve such treatment if patients are not satisfied with the results. The words ‘medical negligence’ is flung around in casual conversations; with poor media coverage adding fuel to fire. These incidents arise from widespread feelings of hopelessness and frustration with the government as well as the medical system that is understaffed and underequipped to handle the patient load in the country.

One notable response from the government is the issuance of an Ordinance on Health workers and Health institutions (First amendment), under article 114 (1) of the Constitution of Nepal on June 6, 2021.^7^ Despite being a landmark decision, it has failed to change the situation due to poor implementation, owing to the political scenario in the country. There is a pressing need for better communication between doctors and patients, improvements in the quality of healthcare services, and health literacy. Doctors need to be better trained to deal with and avoid such situations.^8^ A high standard has to be set for health journalism. Prompt action from police with lawful implementation of the passed ordinance upon reporting incidents of violence is crucial.

It is imperative to recognise that ending violence against doctors is a shared responsibility and it is essential to create a safe workplace environment through prompt and decisive action, for the effective functioning of our already strained healthcare system.

## Contributors

SS conceptualised the study wrote, reviewed, and edited the original manuscript. KP and AK reviewed and edited the original manuscript.

## Declaration of interest

Authors have no conflict of interest to declare.
